# Supplementary material for: Successive Short- and Long-Range Magnetic Ordering in Ba2Mn3(SeO3)6 with Honeycomb Layers of Mn3+ Ions Alternating with Triangular Layers of Mn2+ Ions
Source: Materials (Basel). 2023 Mar 28;16(7):2685. doi: 10.3390/ma16072685 (PMC10095995; doi:10.3390/ma16072685)
Supplement: Supplementary file 1 [file materials-16-02685-s001.zip › materials-2287877-supplementary.pdf]

Supplementary Information

for

Successive Short- and Long-Range Magnetic Ordering in $Ba_2Mn_3(SeO_3)_6$ with Honeycomb Layers of Mn^{3+} ions Alternating with Triangular Layers of Mn^{2+} ions

Artem Moskin [1,2], Ekaterina Kozlyakova [1,2], Seung Hwan Chung [3], Hyun-Joo Koo [3], Myung-Hwan Whangbo [3,4], Alexander Vasiliev [1,2,*]

[1] Department of Low Temperature Physics and Superconductivity, Lomonosov Moscow State University, Moscow 119991, Russia;
[2] Functional Quantum Materials Laboratory, National University of Science and Technology "MISIS", Moscow 119049, Russia
[3] Department of Chemistry and Research Institute for Basic Sciences, Kyung Hee University, Seoul 02447, Republic of Korea
[4] Department of Chemistry, North Carolina State University, Raleigh, NC 27695-8204, USA
* Correspondence: vasil@mig.phys.msu.ru (A.V.); hjkoo@khu.ac.kr (H.-J.K.)

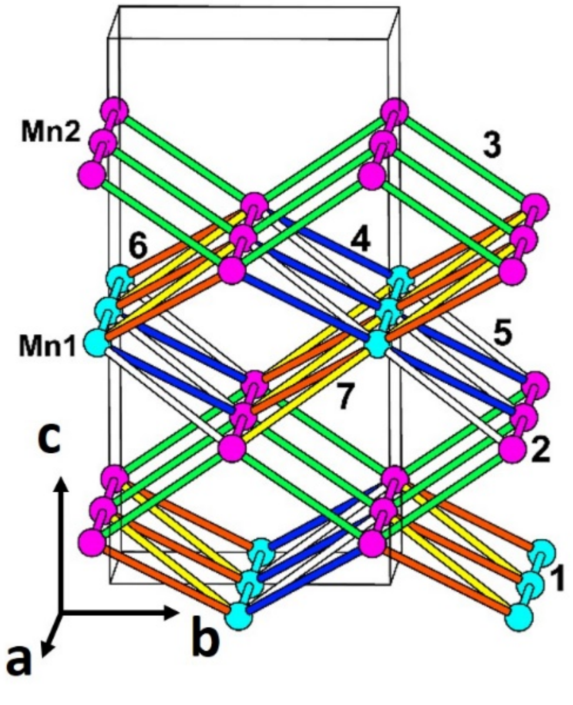

Figure S1. Spin exchange paths in $Ba_2Mn_3O(SeO_3)_6$. Each exchange path is represented by a colored cylinder. The blue and violet circles indicate Mn^{2+} and Mn^{3+} ions, respectively. The numbers 1 – 7 in black represent the spin exchange paths J_1 – J_7, respectively.

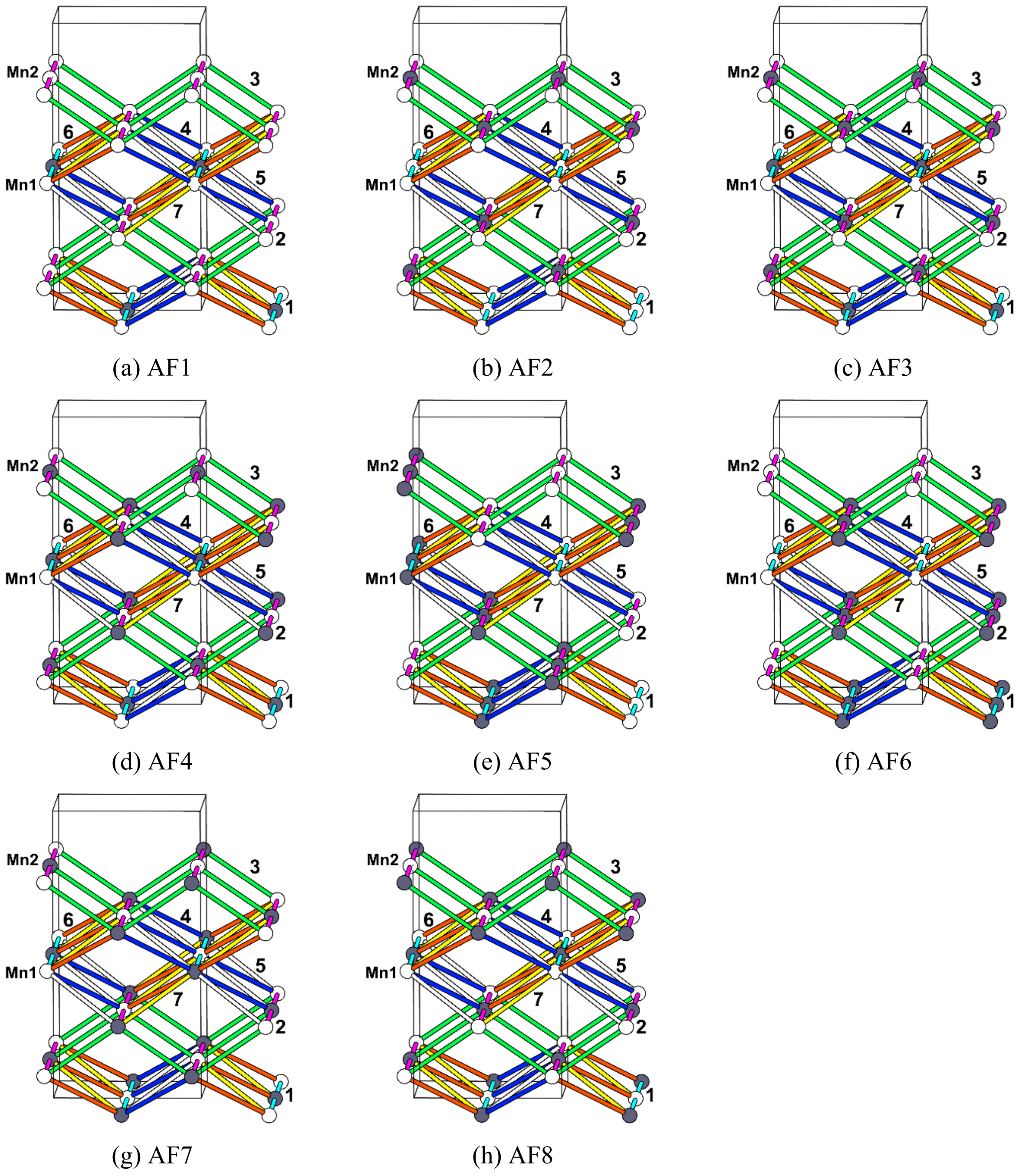

(a) AF1 (b) AF2 (c) AF3

(d) AF4 (e) AF5 (f) AF6

(g) AF7 (h) AF8

Figure S2. Ordered spin states of AF(i) (i = 1 to 8) state. The shaded and open circles indicate the up and down spin sites, respectively.
